# Focus on the role of calcium signaling in ferroptosis: a potential therapeutic strategy for sepsis-induced acute lung injury

**DOI:** 10.3389/fmed.2024.1457882

**Published:** 2024-09-17

**Authors:** Yifei Xu, Xintian Qu, Minghao Liang, Di Huang, Minyan Jin, Lili Sun, Xianhai Chen, Fen Liu, Zhanjun Qiu

**Affiliations:** ^1^First Clinical Medical College, Shandong University of Traditional Chinese Medicine, Jinan, China; ^2^College of Traditional Chinese Medicine, Shandong University of Traditional Chinese Medicine, Jinan, China; ^3^Department of Respiratory, The Affiliated Hospital of Shandong University of Traditional Chinese Medicine, Jinan, China; ^4^Department of Respiratory, Shandong Institute of Respiratory Diseases, The First Affiliated Hospital of Shandong First Medical University and Shandong Provincial Qianfoshan Hospital, Jinan, China

**Keywords:** calcium signaling, sepsis-induced acute lung injury, ferroptosis, iron homeostasis, calcium homeostasis

## Abstract

By engaging in redox processes, ferroptosis plays a crucial role in sepsis-induced acute lung injury (ALI). Although iron stimulates calcium signaling through the stimulation of redox-sensitive calcium pathways, the function of calcium signals in the physiological process of ferroptosis in septic ALI remains unidentified. Iron homeostasis disequilibrium in ferroptosis is frequently accompanied by aberrant calcium signaling. Intracellular calcium overflow can be a symptom of dysregulation of the cellular redox state, which is characterized by iron overload during the early phase of ferroptosis. This can lead to disruptions in calcium homeostasis and calcium signaling. The mechanisms controlling iron homeostasis and ferroptosis are reviewed here, along with their significance in sepsis-induced acute lung injury, and the potential role of calcium signaling in these processes is clarified. We propose that the development of septic acute lung injury is a combined process involving the bidirectional interaction between iron homeostasis and calcium signaling. Our goal is to raise awareness about the pathophysiology of sepsis-induced acute lung injury and investigate the relationship between these mechanisms and ferroptosis. We also aimed to develop calcium-antagonistic therapies that target ferroptosis in septic ALI and improve the quality of survival for patients suffering from acute lung injury.

## Highlights

- Sepsis is the leading cause of death for severely ill individuals. Among the many consequences and multi-organ failure brought on by sepsis, the lung is the most susceptible organ. Ferroptosis, a form of iron-dependent lipid peroxidation-induced programmed cell death, is a key player in the pathophysiology of acute lung injury brought on by sepsis.
- Ca^2+^ is a crucial messenger in cellular life processes. The imbalance between the immune response and inflammation during acute lung injury in sepsis affects intracellular calcium homeostasis, and calcium signaling may accelerate the development of septic ALI. The precise mechanism requires further investigation.
- Abnormal calcium signaling frequently accompanies the process of ferroptosis. The bidirectional interaction of iron homeostasis and calcium signaling may be concurrently implicated in the course of acute lung injury in sepsis. In the realm of critical care medicine, the study on their interaction is still inconclusive.

## 1 Introduction

A severe systemic infection can induce sepsis, a life-threatening visceral organ dysfunction that has a significant socioeconomic impact worldwide. In 2019 there may be a 38% fatality rate from septic shock in Europe and North America (1), according to epidemiologic surveys in China, sepsis can have a mortality rate of up to 40% and a prevalence of up to 25.5% in ICU patients. The lungs account for the largest percentage of infected areas, with a rate of 66% (2). The lung is believed to be the first and most frequently involved target organ in sepsis. About 25–50% of septic patients may develop acute lung injury (ALI) or worse, acute respiratory distress syndrome (ARDS) (3), it manifests as widespread lung inflammation and destruction of alveolar capillaries, eventually leading to alveolar effusion and acute respiratory failure (4). Currently, fluid resuscitation, anti-infection, hemodynamic improvement, mechanical ventilation, and appropriate supportive care are the mainstays of therapeutic treatment for sepsis-induced acute lung injury (5). They are efficient in reducing clinical symptoms in patients, but unintentionally increase the likelihood of bad prognosis (such as immunosuppression, subsequent infections, etc.) (6). In addition, these medications primarily target inflammatory processes and have minimal relevance in restoring programmed cell death. Furthermore, some medications are costly, vulnerable to drug resistance, have major side effects, and must be used for an extended period of time. As a result, there is an urgent need to discover new effective medications, which needs researchers to get a greater understanding of sepsis-induced acute lung injury and examine its pathophysiology.

Ferroptosis is a type of programmed cell death distinct from apoptosis that occurs due to the accumulation of intracellular Fe^2+^ through the Fenton reaction to generate large amounts of Reactive oxygen species (ROS), which oxidize the biofilm and lead to the accumulation of lipid peroxidation products, that play an important role in cancer, ischemia–reperfusion injury, and other multi-system diseases (7). In general, antagonism between the oxidative system consisting of unstable Fe^2+^, Fenton reaction, and ROS as well as the antioxidant system consisting of System Xc^−^, glutathione, and GPX4 maintains the organism in redox homeostasis (7). However, when the body is in the context of infection, inflammation, and immune disorders, the oxidative-antioxidant balance is disrupted, leading to ferroptosis (8, 9). When sepsis occurs, the overwhelming pathogen burden results in the massive production of Lipopolysaccharide (LPS), which induces a storm of inflammatory cytokines, immune regulatory imbalances, and metabolic disorders, resulting in the breakdown of intracellular iron homeostasis, along with the significant intracellular accumulation of Fe^2+^, which promotes lipid peroxidation through the Fenton reaction, the above-mentioned processes are thought to be key mechanisms leading to cell death and poor prognosis in sepsis (10). Ferroptosis has distinct genetically, morphologically, and biochemically properties, and its occurrence is meticulously orchestrated by a finely regulated signal cascade reaction involving multiple components. It is critical to remove superfluous and damaged cells during the illness process. When the ferroptosis regulatory process gets disrupted it can impair normal cells as well as organ function. Current research suggests that ferroptosis is a significant form of programmed cell death in sepsis, and therapies targeting key regulatory molecules of ferroptosis pathways may successfully control acute lung injury in sepsis (11). Numerous clinical and basic studies have demonstrated that inhibition of ferroptosis reduces the extent of lung injury caused by sepsis, For instance, QiShenYiQi pills, a traditional Chinese medicinal formulation, has been shown to exert its anti-inflammatory and antioxidant stress effects by inhibiting ferroptosis and to have therapeutic effects on sepsis-induced acute lung injury (12). Sitagliptin reduces ferroptosis-associated acute lung injury by activating the p62-Keap1-Nrf2 signaling pathway (13). These findings indicate that pharmacologically inhibiting ferroptosis is a promising treatment approach for acute lung injury resulting from sepsis. and Liu et al. proved that the application of ferrostatin-1 significantly attenuates LPS-induced acute lung injury through *ex vivo* experiments (14), Dong et al. found that Astaxanthin, a natural antioxidant, attenuated Fe^2+^ accumulation and lipid peroxidation in lung epithelial cells to mitigate cellular ferroptosis by inhibiting NCOA4-mediated ferritin autophagy (15), Lv et al. identified Protectin conjugates in tissue regeneration 1 (PCTR1) in tissue regeneration to inhibit LPS-induced ferroptosis via the ALX/PKA/CREB signaling pathway to attenuate the septic ALI (16). Currently, research on ferroptosis regulators focuses on experimental animal models, with only a few medications licensed for clinical use. Since it involves dysregulated lipid metabolism, redox balance, and iron metabolism, pharmacological inhibitors of ferroptosis are gaining popularity as a promising avenue for treating ferroptosis-related conditions (17). Fortunately, current research suggests that targeted ferroptosis is a viable treatment option for sepsis-induced acute lung injury. Therefore, it is significant to thoroughly analyze the mechanism of ferroptosis and explore its new targets of influence to save the survival of patients with sepsis-induced acute lung injury.

Ca^2+^ is the second messenger of intracellular signaling and coordinates almost the entire range of cellular processes, calcium signaling can be useful in apoptosis, autophagy, cell proliferation, and cancer regulation (18). Endothelial cell apoptosis is associated with intracellular calcium overload in sepsis-associated pathologies. Changes in intracellular Ca^2+^ levels have been found in different models of sepsis, manifesting as intracellular calcium overload (19, 20). Multiple functions of the apoptotic process are regulated by calcium signaling (21). Recently, research shows calcium overload can give rise to mitochondrial dysfunction and is closely linked to the mechanism of ferroptosis (22). Qiu et al. showed that knockdown of the mouse calcium homeostasis regulation-associated Orai1 and Btk inhibited pulmonary vascular endothelial cell (PVEC) apoptosis in LPS-induced acute lung injury (23). This indicates the emergence of tailored medications with fewer adverse effects and has great promise in the prevention and treatment of sepsis-induced acute lung injury. In this review, we sum up the regulatory mechanism of ferroptosis, reveal its application in sepsis-induced acute lung injury, and highlight the potential role of calcium signaling in ferroptosis, with the goal to provide ideas and references for the mechanism and treatment research of sepsis-induced acute lung injury.

## 2 The ferroptosis regulatory system

### 2.1 Overview of the mechanism of ferroptosis

Ferroptosis is a mode of programmed cell death mediated by iron overload and lipid peroxidation, discovered and named by Stockwell’s group (24). There have been numerous reports on ferroptosis in recent years, and it is now generally accepted that it is mainly characterized by the overloading of Fe^2+^ and the accumulation of lipid ROS, which ultimately leads to oxidative cell death. The process of ferroptosis is ferric-dependent and can be selectively inhibited by iron sequestrants. Three known pathways participate in the buildup of lipid peroxidation products during ferroptosis (25). Firstly, ROS are created through the Fenton reaction. Secondly, ROS are produced by lipid autoxidation, a process that can be regulated by iron-catalyzed enzymatic reactions. Thirdly, ROS are generated by the oxidation of Arachidonic acid (AA) by the Fe^3+^-containing lipoxygenase (LOX) enzymes. The first two pathways are both associated with Fe^2+^. In the Fenton reaction, the combination of Fe^2+^ with hydrogen peroxide produces hydroxyl radicals, whose ability to cause cell death. In addition, the lipid peroxidation products and added Fe^2+^ increase the initiation rate of lipid autoxidation (26). Although Radical-Trapping Antioxidants (RTAs) protect against cellular autoxidation (27), however, when the onset rate of lipid autoxidation exceeds the inherent RTA capacity of the cell, lipid autoxidation happens, leading to cell death. Ferroptosis is governed by a complex network involving iron homeostasis, lipid metabolism, and redox systems. These three viewpoints will be covered in the text that follows.

#### 2.1.1 Mechanisms of iron homeostasis regulation

##### 2.1.1.1 Iron element metabolism in the human body

Iron is the fourth most abundant element in the Earth’s crust, and it is believed that the core of the Earth is composed primarily of iron. Iron is also one of the most abundant essential trace elements in the human body. It is chemically active and can participate in redox reactions by accepting or donating an electron, so it can be involved in many biological processes in the human body, such as DNA synthesis, nucleic acid repair, cellular respiration in the mitochondrion, cell growth, and cell death, host defense and cellular signaling (28). The majority of iron exists in the body in erythrocyte hemoglobin, which is transported to the bone marrow for red blood cell (RBC) production, with a small amount of iron being transported to other tissues for basic cellular processes, and excess iron being transported to the liver for storage. Iron from dietary sources is predominantly in the form of Fe^3+^, mediated by ferric reductases in the apical membrane of enterocytes, such as duodenal cytochrome B. Reduction to the Fe^2+^ form is absorbed into the cytoplasm via divalent metal transporter1 (DMT1), which is expressed in the apical membrane of enterocytes (29), Fe^2+^ is bound to iron-dependent proteins in the cytoplasm or enters the mitochondria and binds to heme or iron–sulfur clusters. Due to the large daily iron requirements of the body, iron is recycled during phagocytosis of senescent erythrocytes by reticuloendothelial macrophages to compensate for the body’s daily iron consumption that is not met by dietary iron levels (28), thereby keeping the body’s iron content in a relatively homeostatic state.

Intracellular transport, delivery, and excretion of iron ions are in relative homeostasis through complex mechanisms that maintain the state of fitness of the body. The mechanisms involved in the regulation of iron homeostasis are mainly the following.

##### 2.1.1.2 The ferric reductase system-STEAP3

STEAP3, a part of the STEAP (Six-transmembrane epithelial antigen of the prostate gene family) gene family, is involved in the regulation of iron homeostasis by acting as an iron ion reductase (30). The STEAP3 protein has been shown to have a transmembrane electron shuttling role, moving NADPH-derived cytoplasmic electrons across the lipid bilayer to the extracellular surface, where they are used to revert Fe^3+^ to Fe^2+^, and functional mutagenesis of the STEAP3 gene results in varying decreases in ferric reductase activity (30). STEAP3 is mainly involved in the transfer of iron from transferrin (Tf). After conjugation with transferrin receptor 1 (TfR1), Tf-TfR1 carries Fe^3+^ through cytosolization. In the nuclear endosome, Fe^3+^ dissociates from Tf-TFR1, which is then transformed to Fe^2+^ by STEAP3 and flows into the cytoplasm under the mediation of DMT1. The dissociated TfR1 then returns to the cytosol surface via cytosolization. The other form of cyclic iron is non-transferrin-bound iron (NTBI), which converts Fe^3+^ to Fe^2+^ via the prion protein (PRNP) with ferric reductase activity, and then mediates the entry of Fe^2+^ into the cell via the zinc transport proteins ZIP8 or ZIP14 (31). Meng et al. found that iron recirculation in lysosomes of mouse macrophage cells is dependent on the STEAP3 reductase enzyme, and showed that constraining repetitive iron cycling by inhibiting STEAP3 expression is an inborn immune reaction that prevents the proliferation of pathogens and sepsis (32).

##### 2.1.1.3 The IRP-IRE regulatory network

The homeostasis of intracellular iron metabolism is majorly modulated by coordinated transcriptional processes through diverse iron metabolism-related genes. Many of the mRNAs involved in encoding ferric metabolism-related proteins contain the iron-responsive element (IRE), which is located in the 5′- or 3′-untranslated region flanking the coding sequence of the stem-loop structure (33). IRE can bind two functionally similar iron-regulatory proteins (IRP), IRP1 and IRP2. The coordinated action of the IRP-IRE network regulates cellular iron uptake, utilization, storage, and export, and increases the bioavailability of iron, resulting in the maintenance of cellular iron homeostasis (34). When iron–sulfur cluster (ISC) synthesis is inhibited, IRP1 and 2 activate the ferric starvation response and increase vulnerability to ferroptosis. Additionally, iron-dependent regulation of IRP2 involves iron sensing by F-box and leucine-rich repeats protein 5 (FBXL5), a substrate-recognition ingredient of the SCFE3 ligase complex, which Wang et al. found to play a vital role in the control of IRP2 recruitment and polyubiquitination in iron homeostasis through the cofactor [2Fe2S] cluster (35). A current study by Terzi et al. found that IRP2 regulates cellular iron homeostasis functionally independent of IRP1 and FBXL5 (36).

##### 2.1.1.4 Ferritin autophagy

Ferritin is the predominant iron-storing protein in mammals and is also critical for maintaining iron homeostasis and preventing the Fenton reaction, the NCOA4-ferritin axis governs intracellular iron homeostasis based on intracellular iron availability, and this process is facilitated by nuclear co-activator receptor 4 (37). NCOA4 is a transcriptional co-activator of the androgen nuclear receptor, and its content in the organism is influenced by iron levels. In iron-sufficient cells, NCOA4 binds to the E3 ubiquitin ligase HERC2 and is degraded by the proteasome, while in iron-deficient cells, NCOA4 combines with ferritin to induce ferritin degradation. In sepsis, LPS increased NCOA4 expression and intracellular Fe^2+^ levels (38). Zhang et al. found that YAP1 alleviated sepsis-induced acute lung injury by inhibiting ferritin autophagy-mediated ferroptosis; Wu et al. found that the interaction between STING and NCOA4 intensifies the lethality of sepsis by modulating ferroptosis in macrophages as well as the proinflammatory effect (39).

##### 2.1.1.5 Hepcidin and ferroportin

Ferroportin (FPN) is the sole intracellular iron exporter, and it is prominently characterized in the regulation of plasma iron concentration. Hepcidin is a hepatic-secreted peptide hormone that exerts an important role by regulating the expression of FPN which is localized on the cell surface. Hepcidin can bind to FPN and induce its internalization as well as degradation in lysosomes, thus suppressing the development of FPN expression. When the circulating iron level surpasses the body’s requirements, hepcidin rises and restrains the expression of FPN, resulting in the repression of iron trafficking from the cells to the circulation. During sepsis, FPN expression is downregulated and its loss-of-function mutations may lead to increased mortality in septic patients. In parallel, fibromodulin maintains iron homeostasis by inducing iron chelation to curtail iron utilization by pathogens, while also inhibiting invasiveness and protecting against sepsis-related organ damage (40). It has been shown that Hepcidin has a higher sensitivity than WBC and CRP in the diagnosis of sepsis in children (41).

##### 2.1.1.6 Inflammatory signaling pathways and iron homeostasis regulation

In the background of sepsis, the functioning of enzymes, hormones, as well as proteins, that participate in the mediation of iron homeostasis requires signaling pathways that interact in a complex manner. Recently, the sophisticated relationship between the activation of inflammatory signaling pathways and disturbances in iron metabolism has attracted attention. Abnormal inflammatory responses are critical for iron homeostasis and redox balance in the body. The pro-inflammatory cytokines, such as IL-1β, IL-6, TNF-*α*, and IFN-*γ*, can modulate the synthesis of ferritin, which influences iron storage in cells and tissues (42). Chen et al. summarized the characterization of ferroptosis in classical inflammatory pathways such as inflammation-associated NF-κB, JAK–STAT, inflammatory vesicles, cGAS-STING, and the MAPK pathway, which are all capable of causing disturbances in intracellular iron metabolism through different mechanisms of iron homeostasis regulation, in particular, the IL-6/JAK/STAT3 modeling pathway, act as a key regulator of hepcidin expression, can cause intracellular iron overload. Furthermore, the imbalance of redox homeostasis in sepsis is also a key component contributing to iron metabolism disorders. It has been found that Nrf2 is the basis of cellular resistance to oxidative damage and the maintenance of redox homeostasis, the Nrf2 knockout mice are more susceptible to LPS-induced acute inflammatory attacks, thus, Nrf2 and its related pathways play an essential part in maintaining iron homeostasis in sepsis. Recently, Anandhan et al. found that Nrf2 can be used to control iron homeostasis and ferroptosis by regulating both HERC2 and VAMP8 (43). In summary, inflammatory signaling pathways are essential segments involved in the maintenance of iron homeostasis in different parts of sepsis (44), deeper exploration of the potential mechanisms of sepsis inflammatory pathways and iron metabolism would be a promising therapeutic direction for studying organ damage in sepsis.

#### 2.1.2 Atypical lipid metabolism

Ferroptosis is characterized by changes in membrane permeability brought on by lipid peroxidation of the cell membrane. Polyunsaturated fatty acids (PUFAs) and membrane phospholipids combine to create peroxides (PUFA phospholipids, PUFA PLs), which can make cells more susceptible to ferroptosis (45). When ferroptosis happens, Arachidonic acid and Adrenic acid in PUFAs are especially vulnerable to peroxidation, which destroys the lipid bilayer (46). An essential process that occurs during ferroptosis is lipid peroxidation, which is catalyzed by acyl-CoA synthetase long-chain family member 4 (ACSL4) and lysophosphatidylcholine acyltransferase 3 (LPCAT3). Enzymes LPCAT3 and ACSL4 are needed for the production and modification of PUFAs. After arachidonic acid or adrenergic acid binds to coenzyme A to produce derivatives AA CoA or Ada CoA, membrane phosphatidylethanolamine is generated, resulting in AA-PE or Ada PE. This esterification reaction is then catalyzed by LPCAT3 (46, 47). Lastly, lipoxygenase promotes ferroptosis by acting subsequent to lipid peroxidation.

Furthermore, various enzymes that produce ROS, including NADPH oxidase (NOX) enzymes, the oxidoreductases cytochrome P450 reductase (POR), NADH-cytochrome b5 reductase (CYB5R1), can generate oxidants that initiate lipid peroxidation and take part in its induction (45, 48, 49). Mitochondria, being the primary sites in cells that produce both ATP and ROS, also contribute to the induction of lipid peroxidation during ferroptosis. The most notable modification in ferroptotic cells is the shift in mitochondrial shape. Studies have demonstrated that energy intake can trigger the activation of AMP-activated protein kinase (AMPK), which phosphorylating and inactivates acetyl-CoA carboxylase, hence effectively inhibiting the synthesis of certain PUFAs and then inhibiting ferroptosis (50, 51). The basic mechanism of ferroptosis is lipid peroxidation, which is a dynamic process regulated by various substances, enzymes, etc. Treatment for ferroptosis may depend on greater investigation into the mechanisms of lipid uptake, storage, metabolism, and remodeling during the disease. This suggests that targeted medications are becoming more prevalent and warrants more research.

#### 2.1.3 Imbalance of redox system

**Figure 1:**
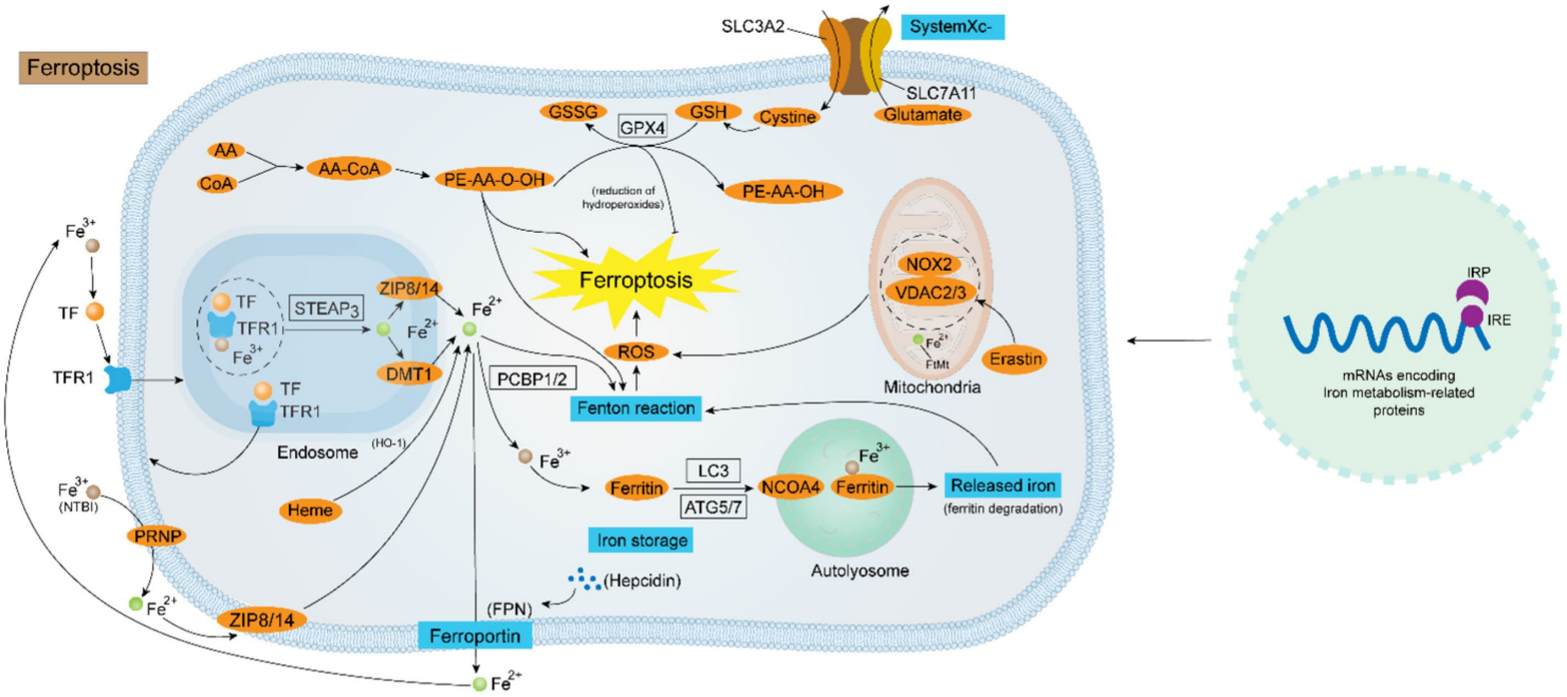
Diagrammatic representation of the ferroptosis regulating mechanism. Iron homeostasis is a sophisticated kinetic process regulated by various factors at the cellular level. At the genetic level, the collaboration of the IRP-IRE system is critical for the maintenance of intracellular iron homeostasis. At the molecular level, the uptake of Fe^2+^ is mediated by the TF-TFR1-STEAP3-DMT1-ZIP8/14 system or via the PRNP-ZIP8/14 pathway flowing into the cell. NCOA4-mediated ferritin autophagy is also a route for intracellular Fe^2+^ accumulation. The output of Fe^2+^ is instead controlled by both Hepcidin and FPN. Intracellular Fe^2+^ overload is the key to the occurrence of ferroptosis. Firstly, Fe^2+^ generates a large amount of ROS through the Fenton reaction, and ROS oxidizes the biofilm causing ferroptosis; secondly, the disruption of the antioxidant system mediated by System Xc^−^ - GPX4 causes ferroptosis; thirdly, some ROS-generating enzymes (e.g., ALOXs, POR, and NOX), and channels in the mitochondrial membrane (e.g., VDAC2/3) also contribute to the ferroptosis progression.

On the other hand, the disruption of the anti-oxidant regime is also an implicated mechanism in the occurrence of ferroptosis, which is mainly reliant on the regulation of the SystermXc^−^-GSH-GPX4 pipeline. SystermXc^−^ is an amino acid antitransporter protein composed of two key subunits, SLC7A11 and SLC3A2, that normally mediates the exchange of cystine and glutamate across cell membranes. Once cystine enters the cell then is utilized to synthesize glutathione, subsequently, GPX4 can use the synthesized glutathione to detoxify the lipid peroxidation products generated by cellular oxidative stress, which is the body’s primary antioxidant mechanism. Studies have shown that many ferroptosis inducers (e.g., Erastin) can degrade GPX4, leading to the inactivation of the antioxidant system, which results in the onset of ferroptosis. Moreover, when tumor suppressor protein p53 is over-activated, the expression of SLC7A11, a component of the SystermXc^−^, is suppressed, thereby exacerbating the disruption of the antioxidant defense system (52). Recently, a GPX4-independent antioxidant pathway was identified in ferroptosis that relies on AIFM2-mediated coenzyme Q (CoQ) production or GTP cyclic hydrolase 1 (GCH1)-regulated tetrahydrobiopterin (BH4) synthesis (53). Coenzyme Q, also called ubiquinone, and coenzyme Q10 are potent lipophilic antioxidants that play important roles in the mitochondrial respiratory chain, as AIFM2 inhibits ferroptosis by catalyzing the production of CoQ2H10 from CoQ10 or by promoting ESCRT-III-dependent membrane repair. Restoring intracellular redox equilibrium is one of the most important steps in preventing ferroptosis, which means that figuring out the mechanism underlying the antioxidant system’s malfunction in ferroptosis is an essential therapeutic option (Figure 1).

## 3 Ferroptosis’s involvement in sepsis-induced ALI

Iron serves a vital part for sustaining human life activities. Nevertheless, too much iron increases the generation of reactive oxygen species and lipid peroxidation products, which are detrimental to the host and cause cell toxicity. Lipid peroxidation and iron overload are characteristics of ferroptosis for the same reason. An increasing amount of data indicates that iron overload encounters a role in the emergence and progression of acute lung injury in sepsis (54). For instance, ferroptosis inhibitors (Ferrostatin-1) enhanced cell survival and greatly decreased the total iron concentration of LPS-induced bronchial epithelial cells (BEAS-2B)*in vitro* (14). This suggests that iron excess has a detrimental influence on sepsis-induced acute lung injury. Consequently, the following article provides an overview of how ferroptosis affects cell death and structural damage to alveoli during acute lung injury in sepsis.

The alveoli are the main site of gas exchange and the functional unit of the lung. The alveolar epithelium serves as a technologic shield that prevents alveolar damage and consists primarily of type I and type II alveolar epithelial cells, which are actively involved in the immune response of our lungs and contribute to the maintenance of alveolar superficial fluid homeostasis. The alveolar epithelium is in tight proximity to the endothelial monolayer of the pulmonary capillary reticulation, and alveolar macrophages (AMs) roam the alveolar lumen and the vicinity of the endothelial cells of the capillaries, and the interstices between these two types of cells contain fibroblasts (55). Under normal conditions, the pulmonary epithelial barrier and the pulmonary vascular endothelial barrier, as well as the major cells in these barriers, constitute the homeostatic environment of our lungs. However, in the setting of LPS-induced sepsis, these cells and the barriers they form cause varying degrees of disruption (56). Sepsis causes damage to alveolar epithelial cells and capillary endothelial cells in the lung tissue by generating inflammatory cells to proliferate and activate within the lung tissue. As the susceptibility of pulmonary capillaries to protein molecules grows, and exchange of fluid is obstructed, leading to osmosis pulmonary edema.

The primary pathophysiological reasons for sepsis-induced acute lung injury are alveolar capillary breakdown and widespread pulmonary inflammation. The collapse of alveolar structure and loss of function requires the depletion of alveolar epithelial cells (AE1 and AE2). In alveolar epithelial cells, ferroptosis is a result of intricate regulatory processes. For instance, anomalies in the Hepcidin and Ferroportin system can result in iron overload in sepsis, which damages the alveolar septa’s ultrastructure and causes membrane-bound iron deposition in AE1 cells, thickening of the gas blood barrier, and proliferation and atrophy of AE2 cells (57); Alveolar epithelial cells’ mRNA level of the RNA binding protein AU rich element RNA binding factor 1 (AUF1) decreases in response to ferroptosis inducers. The proteasome pathway is used by E3 ubiquitin ligase FBXW7 to ubiquitinate AUF1, which in turn promotes AUF1 protein degradation during ferroptosis. Concurrently, AUF1 facilitates the degradation of ATF3 mRNA and maintains the stability of NRF2 mRNA, both of which are critical for AUF1-mediated resistance to ferroptosis (58). Zhang et al. revealed that neutrophil extracellular trap (NET) promotes the progression of ALI in sepsis by inducing ferroptosis in alveolar epithelial cells (59). Wang et al. discovered that the Hippo signaling pathway causes alveolar epithelial cells to undergo ferroptosis in response to acute lung injury when exposed to the extracellular vesicle tRF-22-8BWS7K092 produced from alveolar macrophages (60).

The pulmonary microvascular endothelial barrier is a semi-selective physical and immunologic barrier composed of Pulmonary Microvascular Endothelial Cells (PMECs) that controls the exchange of fluid and solutes in and out of pulmonary vessels (61). The excessive inflammatory response during sepsis leads to large-scale destruction and increased permeability (62), this pathologic stage is known as the exudative phase and belongs to the initial phase of ALI (63). One of the main mechanisms underlying the disintegration of the pulmonary microvascular endothelial barrier and aggravation of ALI during sepsis is ferroptosis of pulmonary microvascular endothelial cells. Primary mouse pulmonary microvascular endothelial cells used in *in vitro* tests demonstrated increased levels of lipid peroxidation products (malondialdehyde, MDA) and (4-hydroxynonenal, 4HNE), decreased levels of GPX4, and more severe cases of acute lung injury. According to further research, the Keap1/Nrf2/GPX4 pathway in adipose-derived stem cell exosomes mitigates the iron mortality of pulmonary microvascular endothelial cells in septic ALI (64). According to a study conducted on human pulmonary microvascular endothelial cells, exposure to LPS enhanced STEAP1 expression, which in turn increased pro-inflammatory cytokines including IL-1β, IL-6, and adhesion molecules like ICAM-1, hence intensifying the severity of ALI. Its mode of action involves both an increase in ROS levels and the suppression of intracellular antioxidant molecules Nrf2 and GPX4 (65).

**Figure 2:**
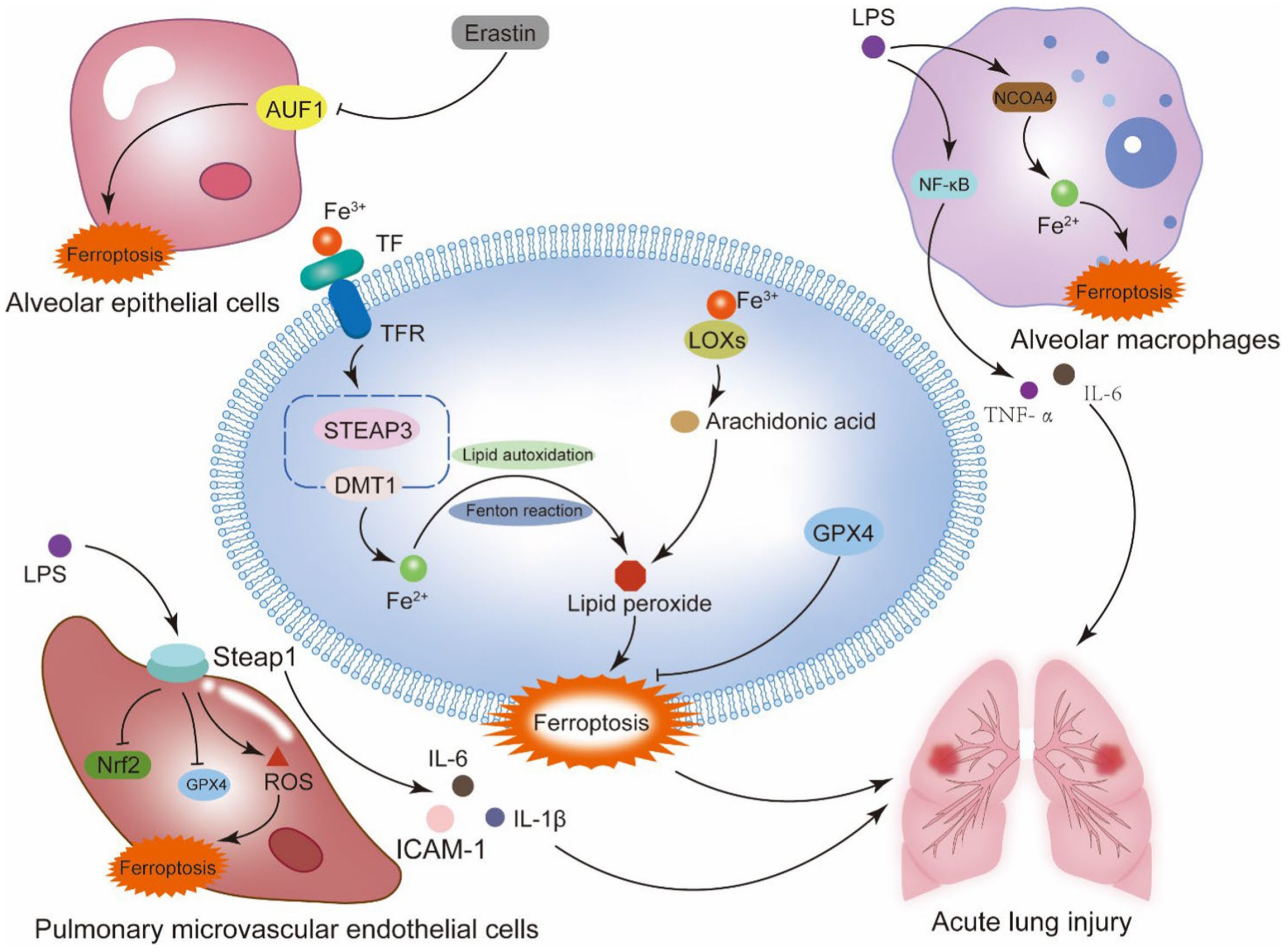
Ferroptosis and sepsis-induced acute lung injury. Alveoli are the main site of lung gas exchange and the functional unit of the lung. The alveolar epithelium is a mechanical barrier that protects the alveoli from injury, and the alveolar epithelial barrier and the vascular endothelial barrier work together to provide a homeostatic environment for the lung. This system contains type I alveolar epithelial cells, type II alveolar epithelial cells, alveolar macrophages, pulmonary microvascular endothelial cells, and fibroblasts. Ferroptosis of the cells that make up the alveolar epithelium and vascular barrier is a key mechanism of septic ALI. The generation of lipid peroxidation products and the disruption of the antioxidant system in these cells are the primary mechanisms behind ferroptosis. In alveolar epithelial cells, the RNA binding protein AU rich element RNA binding factor 1 (AUF1) influences the mRNA stability of two ferroptosis regulating factors, NRF2 and ATF3. By positively regulating NRF2 and negatively regulating ATF3, AUF1 prevents ferroptosis. In pulmonary microvascular endothelial cells, the suppression of Nrf2 and GPX4 by STEAP1, as well as the generation of pro-inflammatory cytokines, may be the primary processes worsening sepsis-induced ALI. In alveolar macrophages, NCOA4-mediated autophagy of ferritin causes intracellular iron overload, aggravating sepsis-induced ALI.

Alveolar macrophages are large mononuclear phagocytes, including two types of subtypes, M1 and M2, which have opposite pro-inflammatory and anti-inflammatory functions, Macrophages perform an essential part in maintaining iron homeostasis, and an accumulation of iron within the cell can cause M1 macrophages to become polarized. Research has shown that LPS is a classical activator of AM, which binds to Toll-like receptor 4 to activate the NF-κB pathway, releasing pro-inflammatory mediators IL-6, TNF-*α*, etc., resulting in clinical manifestations of lung tissue injury and sepsis (56). Studies have indicated a connection between ferroptosis and when ferroptosis occurs to macrophages, which can release several inflammatory cytokines. Recent studies have shown that ALI is linked to increased ferritin autophagy by NCOA4 in alveolar macrophages, which raises Fe^2+^ overload and ARDS aggravation that melatonin can reduce (66). In summary, the mechanisms underlying ferroptosis’s involvement in the immunological, inflammatory, and cell death processes of sepsis-induced acute lung injury are highly complex. However, this also provides a mode of action for the synthesis of several target drugs. It is a difficulty and a chance for the ALI drug investigation and mechanism (Figure 2).

## 4 Relationship between calcium homeostasis and ferroptosis

### 4.1 Intracellular regulation of calcium homeostasis

Within the cell, Ca^2+^ exists in three predominant forms: storage calcium, conjugated calcium, and free calcium. The concentration of free Ca^2+^ in the cytoplasm is at a relatively low level, maintained at about 100 nM in the resting state, to ensure effective cell signaling and prevent cytotoxic effects, which is referred to as calcium homeostasis. Calcium homeostasis reflects the central significance of Ca^2+^ as a second messenger to the cell, which is involved in regulating cell proliferation and differentiation, regulatory cell death, gene transcription, metabolism, neurotransmission, and other essential functions (67). Calcium ions that exist in the cytoplasm come from two main sources: the extracellular inward flow of Ca^2+^ and the intracellular release of stored Ca^2+^. Calcium homeostasis is regulated and maintained by several calcium transport systems in cell membranes and organelle membranes, and it exists as a complex system of hormones, Ca^2+^ transporter proteins, calcium channels, exchangers, binding or buffering proteins, and Ca^2+^ pumps, which fine-tunes the Ca^2+^ mobility within and outside the cell as well as across diverse organelles (68).

Extracellular calcium endocytosis is mainly an active mode of transport, there are multiple Ca^2+^ channels present in the cytoplasmic membrane, comprising the transient receptor potential (TRP), voltage-gated calcium channels (VGCC), store-operated Ca^2+^ entry (SOCE) channels, calcium-sensing receptor (CaSR), Na^+^/Ca^2+^ exchanger (NCX), as well as the protein misfolding cyclic amplification (PMCA) or ATP2B, which are considered as the major calcium homeostatic maintainers in the cytomembrane, they transport Ca^2+^ into the cell against the concentration gradient by consuming ATP.

Nevertheless, the maintenance of intracellular calcium homeostasis closely relates to two essential cell organs, the mitochondria and the endoplasmic reticulum. Mitochondrial calcium homeostasis is maintained through three main pathways, mitochondrial calcium uniporter (MCU), sodium/calcium/lithium exchange protein (NCLX), and calcium-hydrogen exchange protein (CHE), in addition to fast-mode calcium uptake and ryanodine receptors, which are also thought to play an important role under certain conditions. Mitochondria have a unique ability to accumulate large amounts of calcium in their matrix, and pathological states of calcium overload can also affect mitochondrial functions such as the TCA cycle and the electron transport chain (ETC), inhibiting mitochondrial ATP synthesis and causing mitochondrial ultrastructural damage (69). Similarly, the endoplasmic reticulum, the predominant Ca^2+^ reservoir in mammalian cells, exists in a steep gradient with calcium concentrations in cytoplasmic lysate and mitochondria, which are maintained at about 100 uM to 1 mM (70). Endoplasmic reticulum Ca^2+^ homeostasis is tightly regulated in both directions by cytoplasmically derived Ca^2+^ uptake and Ca^2+^ leakage channels. Among them, inositol 1,4,5-trisphosphate receptors (IP3Rs) and Ryanodine receptors (RyRs) are the major endoplasmic reticulum calcium leakage channels (70). Dysregulation of endoplasmic reticulum Ca^2+^ content affects problematic protein folding, leading to the accumulation of misfolded proteins in the endoplasmic reticulum and inducing endoplasmic reticulum stress (ER stress). IP3R-mediated Ca^2+^ release also triggers the release of several pro-apoptotic factors from mitochondria and activates apoptosis. In parallel to this, Ca^2+^ signaling also occurs between the endoplasmic reticulum and mitochondria. This requires the close juxtaposition of the endoplasmic reticulum membrane and the mitochondrial outer membrane, thereby forming microstructural domains, which are named mitochondria-associated endoplasmic reticulum membranes (MAMs). MAM contacts with widths ranging from 10 to 25 nm are optimal for Ca^2+^ transfer, the so-called “Ca^2+^ hot spots.” In these regions, Ca^2+^ concentrations are very high and reach levels of more than 10 μM (71). As previously stated, dynamic Ca^2+^ fluxes between various organelles and intracellular Ca^2+^ concentration are strictly regulated, and calcium homeostasis preservation is essential for intracellular redox homeostasis and ATP levels.

### 4.2 Calcium homeostasis takes part in the physiological process of ferroptosis

Calcium signaling plays an essential role in several forms of cell death, such as apoptosis, autophagy, and pyroptosis (72). Here, we focus on the possible engagement of calcium signaling in the mechanism of ferroptosis, in which we suggest that Ca^2+^ is involved in the process of ferroptosis directly or indirectly, intracellular calcium overload mediates the massive production of ROS, which aggravates lipid peroxidation and the disruption of antioxidant systems. At the same time, there is bidirectional crosstalk between Ca^2+^ and Fe^2+^, with calcium overload increasing intracellular levels of unstable Fe^2+^ and stimulating ferroptosis caused by iron overload.

#### 4.2.1 Ca^2+^ participates in ferroptosis by regulating ROS production

Iron has a strong oxidizing ability, in acidic nuclear endosomes, Fe^3+^ is converted to Fe^2+^ and then transported to the cytoplasm through divalent metal transporter 1 (DMT1). During the process of acute lung injury, LPS disrupts iron homeostasis resulting in iron overload, the excessive Fe^2+^ generates a large amount of reactive oxygen species (ROS) through the Fenton reaction, thus triggering the oxidative stress of sepsis, which is called lipid peroxidation, and thus causing ferroptosis. In this procedure, ROS mediates the correlation between Ca^2+^ ferroptosis.

Intracellular calcium signaling manipulates ROS levels by targeting the major ROS-generating sites, including mitochondrial respiration and NOX enzymes (73), and keeping intracellular ROS levels under control and maintaining them are vital parts of ferroptosis. For instance, Ca^2+^ enhances the activity of Krebs cycle dehydrogenase, which affects ATP synthase, adenine nucleotidyltransferase (ANT), and glycerol-3-phosphate dehydrogenase to regulate the production of mitochondrial ROS. Miao et al. reported that Chlorpyrifos (CPF)-mediated intracellular calcium overload exacerbated the production of mitochondrial ROS, thereby aggravating cell death (74). Ca^2+^ also can regulate the activity of NOX_2_, which is the main enzyme that produces ROS, and calmodulin-dependent kinase II (CaMKII) has the effect of activating NOX_2_, and Ni et al. found that its inhibitor, KN-93, significantly downregulated the expression of NOX_2_ and NOX_4_, which significantly decreased the level of mtROS (75).

Moreover, ROS stimulates an increase in cytoplasmic Ca^2+^ influx. During sepsis, LPS stimulates cellular oxidative stress to generate ROS, which is triggered by the modulation of InsP3Rs, SERCA2B, TPCs, SOCE, and several TRP channel subtypes to Endothelial cell Ca^2+^ signaling (76). Early studies have identified a TRPM family member, TRPM2, which bridges ROS and Ca^2+^ influx due to the unique NUDT9-H structural region of TRPM2 at its C-terminus, which senses ADPR and shares similarities with the mitochondrial NUDT9 enzyme. During oxidative stress associated with ferroptosis, TRPM2 converts ROS signaling into Ca^2+^ influx, which in turn is accelerated by intracellular calcium overload (77). It was found that TRPM2 mediates the oxidative burst of neutrophils in the immune system (78), and Yamamato and his colleagues showed that silencing TRPM2 blocked H_2_O_2_-induced Ca^2+^ influx and the downstream Ca^2+^-dependent tyrosine kinase Pyk2 (79). It was also reported that TRPM2-mediated Ca^2+^influx was involved in LPS-induced primary production of several cytokines (IL-6, IL-8, IL-10, and TNF-*α*) in monocytic THP-1 cells (80). This indicates that cellular redox imbalance and inflammatory damage are linked to an increase in Ca^2+^ influx, and that there is an interaction relationship between ROS and Ca^2+^ crosstalk. One potential strategy to intervene in ferroptosis is to concentrate on the regulatory effect of Ca^2+^ on intracellular ROS levels.

#### 4.2.2 Ca^2+^ participates in ferroptosis by disrupting the cellular antioxidant system

Physiologically, cells can detoxify biofilm lipid peroxides, relying primarily on the glutathione peroxidase 4 (GPX4), which reduces lipid peroxides to lipids alcohols with the aid of the reductant glutathione (GSH), thereby mitigating ferroptosis (81). Lipid peroxidation is a hallmark of cellular ferroptosis, and numerous studies have shown that GPX4 inactivation induces ferroptosis in different cells with nonmitochondrial lipid peroxidation. Ca^2+^ has a role in inhibiting the expression of molecules associated with the GPX4 system. Silvia et al. found that endoplasmic reticulum Ryanodine receptor-mediated Ca^2+^ release helps to restore the primary hippocampus that occurs as a result of GPX4 inhibition ferroptosis in neurons (82). RyR is an ion channel localized in the membrane of the cellular sarcoplasmic reticulum/endoplasmic reticulum, which functions to release Ca^2+^ stored in the sarcoplasmic reticulum/endoplasmic reticulum into the cytosol upon cellular stimulation, triggering the biological process of Ca^2+^ activation. PIEZO1, a mechanically-activated, osmotic calcium ion channel, is highly expressed in pulmonary tissues, and a study by Guo et al. revealed that ferroptosis was accompanied by a decrease in the expression of GPX4 and SLC7A11 and an increase in the expression of DMT1 (83), this implies that intracellular calcium overload, which promotes ferroptosis in pulmonary tissues caused by GPX4 depletion, while increased DMT1 expression facilitates intracellular Fe^2+^ translocation, indicating that intracellular calcium homeostasis has unique significance for the progression of ferroptosis in lung tissue.

#### 4.2.3 Ca^2+^ participates in ferroptosis by mediating mitochondrial dysfunction and endoplasmic reticulum stress

Nearly all eukaryotic cells have mitochondria, which are organelles. When mitochondria undergo oxidative phosphorylation to make ATP, reactive oxygen species (ROS) are produced. Depending on the internal environment of the mitochondria, ROS can be advantageous or harmful. Through a decrease in antioxidant defenses and an increase in oxidative stress, sepsis can lead to mitochondrial dysfunction. While the buildup of ROS and lipid peroxidation products can worsen mitochondrial damage, mitochondrial dysfunction can also encourage the overproduction of ROS (84). Apart from oxidative phosphorylation, mitochondria serve as intracellular calcium ion reservoirs. The TCA cycle is a Ca^2+^-dependent activity, and mitochondrial function and Ca^2+^ dynamics are interwoven processes (85). The absorption of calcium ions by mitochondria occurs via two primary routes. The first is the cytoplasmic absorption of calcium ions. The mitochondrial calcium uniporter (MCU), mitochondrial ryanodine receptor Type 1 (mRYR1), mitochondrial permeability transition pore (mPTP), and others are examples of the mitochondrial Ca^2+^ channels found in the ionically hypo-permeable inner mitochondrial membrane (IMM) (86). One of the MCU complex’s components, MICU1, serves as a gatekeeper in this instance by establishing a threshold that prevents mitochondrial calcium uptake when cytoplasmic Ca^2+^ falls below normal. The activity of mitochondrial MCU is linked to Fe^2+^ loading, and iron overload causes the formation of ROS and membrane potential depolarization in the mitochondria, which opens the mPTP and promotes calcium signaling. By preventing ferroptosis, inhibition of mitochondrial MCU may mitigate heart dysfunction brought on by iron excess. This suggests that Fe^2+^ levels during ferroptosis affect Ca^2+^, and that an increase in calcium flow can subsequently encourage oxidative stress and mitochondrial malfunction. Ferroptosis is connected to the fatal synergistic connection between Ca^2+^ and Fe^2+^ (87).

The endoplasmic reticulum is the largest organelle in eukaryotic cells and consists of two types, the rough endoplasmic reticulum and the smooth endoplasmic reticulum. ER performs a variety of cellular functions such as protein folding, transport and modification, calcium storage, lipid synthesis, and carbohydrate metabolism (88). In some pathological states, some proteins in the ER fail to fold correctly and naturally, leading to the aggregation and accumulation of unfolded proteins in the lumen of the ER, a condition known as ER stress (89). In inflammatory diseases, endoplasmic reticulum stress can trigger infection by promoting TLR4-stimulated macrophage secretion of the interleukin IL-1β via caspase-8- and TRIF-dependent pathways, as well as by participating in the activation of NLRP3 inflammasome vesicles (90). In septic ALI, endoplasmic reticulum stress is a corollary of the inflammatory response with overactivation of oxidative stress. Chen et al. found that heme oxygenase 1 could inhibit ER stress and reduce apoptosis in the lung following sepsis by inhibiting the PERK/eIF2-*α*/ATF4/CHOP pathway (91). Ferroptosis is a critical factor in septic ALI and is regulated by ER stress. Zeng et al. found that rmMANF prevented septic ALI by inhibiting endoplasmic reticulum stress-induced ferroptosis (92). The endoplasmic reticulum serves as a reservoir for intracellular calcium ions and most endoplasmic reticulum-associated proteins are involved in maintaining endoplasmic reticulum calcium homeostasis. For example, SERCAs, RyR, IP3R and Bip. During sepsis, endoplasmic reticulum stress disrupts calcium homeostasis, leading to cellular calcium overload and oxidative stress, which is a potential pathogenesis of sepsis (93). Subsequent investigations have revealed that ferroptosis can be mediated by endoplasmic reticulum stress via the PERK-Nrf2-HO-1 pathway (94). In the endoplasmic reticulum, PERK is a calcium homeostasis regulator that controls SERCA activity to preserve calcium homeostasis (95). Ma et al. showed that the presence of Wnt5a recombinant protein induced endoplasmic reticulum stress and ferroptosis by increasing the endoplasmic reticulum stress proteins Atf6, Chop, and the nuclear transcription factor of the Ca2+ signaling pathway, Nfat1, as well as decreasing the markers of ferroptosis, Gpx4, and Slc7a11 (96).

#### 4.2.4 Ca^2+^ participates in ferroptosis by targeting the regulation of iron homeostasis

##### 4.2.4.1 Abnormal calcium signals excited by iron overload

Septic ALI destroys iron homeostasis, and one of the many adverse consequences of iron overload is the stimulation of aberrant intracellular calcium signaling, which has secondary effects on a range of cellular life processes. The uptake of Fe^2+^ can be mediated by the plasma membrane transport protein DMT1, whereas Fe^3+^ accumulates mainly through TFRs in the form of TF binding. Guan et al. demonstrated experimentally that the overloading of Fe^2+^ and Fe^3+^ in astrocytes triggers an increase in intracellular Ca^2+^ endocytosis through two different signaling cascades, independently. The cytoplasmic Na^+^ concentration rises as a result of the uptake of Fe^2+^ by DMT1’s inhibition of the Na^+^-K^+^-ATPase, reversing the Na^+^ and Ca^2+^ exchange in the sodium-calcium exchanger (NCX). This results in a rapid and prolonged plateau phase of the inward flow of Ca^2+^, with little to no decrease in Ca^2+^ levels in the presence of Fe^2+^ overload. Reversal of the Fe^3+^-TF-TFR translocation, PLC activation, and the release of InsP3 cause the accumulation of Fe^3+^. This causes an increase in endoplasmic reticulum Ca^2+^ release, which is momentarily increased and recovers to baseline in 200–300 s (97). It is evident that intracellular iron and calcium homeostasis are closely related, and that the excess iron levels in septic lung injury may be critical in triggering aberrant calcium signaling, this issue deserves further in-depth exploration.

##### 4.2.4.2 The iron overload mediated by the calcium signaling

In pathological conditions, calcium overload can prompt several calcium channels and calcium-dependent enzymes located in the membrane and organelle membrane to exert effects that can mediate the inward flow of Fe^2+^. Two types of voltage-gated calcium channels (VGCCs) that generate pores and are multisubunit include L-type calcium channels (LTCCs) and T-type calcium channels (TTCCs). These channels can open in response to membrane depolarization, letting Ca2+ enter the cell. Fe2+ influx into the cell can be mediated by both LTCCs and TTCCs (98). According to Wu et al., intracellular Fe2+ levels can rise due to calcium overload, which is mediated by endoplasmic reticulum RyR and plasma membrane L-type calcium channels. This causes ferroptosis in microglia (99). According to Zhang et al., ferroptosis is linked to the Ca^2+^-dependent activation of PKC isoforms, and the effect of ferroptosis is amplified via the lipid peroxidation-PKCβII-ACSL4 positive feedback axis (100). Chen et al. found that the anti-lung cancer effect of Erianin may be through the activation of the Ca^2+^-calmodulin (CaM) pathway, which regulates L-type voltage-dependent Ca^2+^ channels (LVDCC) and promotes its iron absorption to induce ferroptosis (101). In the study by Sun et al., controlling calcium homeostasis might successfully prevent neuronal cell ferroptosis and be a promising new target for Alzheimer’s disease treatment (102). Additionally, several clinical studies have demonstrated the potential effects of calcium channel blockers on myocardial iron levels, showing a 27% reduction in myocardial iron concentration measured by MRI in patients treated with the calcium antagonist amlodipine for 1 year (103). Amlodipine treatment significantly reduced myocardial iron concentrations in participants with higher than normal levels by 21%, while the placebo-treated control group showed no change in myocardial iron concentrations (104). These investigations have shown how calcium signaling controls the uptake and content of iron ions *in vivo*. This suggests that calcium channel blockers or calcium antagonists may be able to lower intracellular iron levels and prevent ferroptosis. Uncovering the mechanism of the calcium-iron relationship in the context of critical care is of enormous breakthrough significance, but there are still few comprehensive mechanistic investigations in the field of septic ALI.

## 5 Evidence of the significance of calcium signaling in acute lung injury caused by sepsis

### 5.1 The possible medicinal benefits of regulating calcium homeostasis in severe disorders

The imbalance of calcium homeostasis, which often occurs in sepsis patients, is thought to be pivotal in mediating organ dysfunction in sepsis, and the crosstalk between aberrant calcium signaling and inflammation is involved in the progression of sepsis organ damage through intricate mechanisms. Hypocalcemia often occurs in sepsis patients, and this is associated with a decrease in calcium levels mediated by the calcium-sensitive receptor (CaSR), which regulates calcium homeostasis, in response to pro-inflammatory signals such as IL-1β, IL-6, and others (105). The inward flow of Ca^2+^ may be the main trigger for the development of hypocalcemia, which is associated with disruption of cell membranes by pathogenic microorganisms and increased permeability of cell membranes to Ca^2+^. Nonetheless, although it is common for systemic calcium levels to decrease during sepsis, intracellular calcium overload is manifested. By triggering the release of calcium from the endoplasmic reticulum and activating store operated calcium channels and transient receptor potential (TRP) channels, activation of calcium sensitive receptors (CaSR) increases intracellular calcium levels and stimulates calcium influx into cells (106). Sepsis and viral infections like COVID-19 can be effectively treated by regulating calcium homeostasis, which has grown in importance as a potential therapeutic approach for critical conditions. It has been established that the elevated levels of intracellular Ca^2+^ in pulmonary microvascular endothelial cells can contribute to the progression of ALI by increasing endothelial permeability and exacerbating the inflammatory response. Such as reducing the VE-cadherin-dependent sites of cell adhesion (107, 108). Zhang et al. demonstrated that calcium binding to CAM leads to CAMK activation, which is a novel inflammatory pathway. Activation of CAMK4 is involved in the activity of NLRP3 inflammatory vesicles in the upstream pathway of LPS-induced acute lung injury (109), which supports the engagement of calcium signaling in the excitation process of septic ALI. Peng et al. found that significant calcium overload was observed in septic lung tissue and that blocking cADPR-mediated calcium overload remarkably ameliorated lung injury (110). Since the COVID-19 pandemic, viral sepsis has emerged as a challenging problem for intensive care units. Studies has revealed that viruses can induce intracellular calcium overload and take part in multiple biological processes, including the fusing of viral particles into host cells, the creation of virus proteins, the maturation of viruses, and their release (111). Increased intracellular calcium influx activates pro-inflammatory pathways such NLRP3 and NF-κB, resulting in systemic inflammatory response syndrome (106, 112). Consequently, calcium channel blockers have been shown to be beneficial against viral infections such as influenza A virus, Japanese encephalitis virus, hemorrhagic fever sand virus, and Ebola virus, as well as calcium homeostasis regulators have been suggested for use in viral infections and sepsis (113, 114). Collage et al. proposed that calcium supplementation during sepsis can exacerbate organ failure and mortality through calcium/calmodulin-dependent protein kinase kinase (CaMKK) signaling, whereas calcium antagonism may contribute to the recovery of cellular functions (115). Therefore, targeting the inhibition of calcium influx into cells is an effective therapeutic strategy for septic ALI. Currently, however, there is still a lack of research on the mechanism of calcium overload in the field of septic ALI, which remains a direction for further investigation in the future.

### 5.2 “Calcium induced calcium release” is the cause of septic ALI

The pulmonary microvascular endothelial barrier is a semi-selective physical and immunologic barrier composed of Pulmonary Microvascular Endothelial Cells (PMECs) that govern the exchange of liquids and solutions inside and outside the pulmonary vasculature (61). The excessive inflammatory response that occurs during sepsis causes it to become large-scale disrupted and hyperpermeable, this pathological phase is known as the exudative phase and belongs to the initial stage of ALI. It has been found that calcium inward flow and calcium signaling cascades mediate septic ALI by disrupting the pulmonary vascular endothelial barrier and endothelial cell death (107). Then, how does sepsis lead to intracellular calcium overload?

Transient Receptor Potential Vanilloid 4 (TRPV4), belongs to the TRPV subfamily and is a non-selective cation channel that is commonly expressed in a variety of cells including endothelial cells, epithelial cells, and macrophages (116). TRPV4 has six transmembrane structural domains similar to those of other TRP proteins, with an ion channel pore between the fifth and sixth transmembrane structural regions that allow the passage of a wide range of cations, and is a non-selective, mechanosensitive, transmembrane Ca^2+^-permeable cation channel. TRPV4 can be activated in response to a wide range of biochemical and biomechanical stimuli including mechanical deformation, osmotic stimuli, thermal stimuli, and endogenous and exogenous chemical stimuli (117). In bronchial epithelial cells, TRPV4 can be activated independently of the classical host pattern-recognition receptor TLR4 in response to LPS stimulation, inducing Ca^2+^ influx (118), the same phenomenon also exists in urothelial epithelial cells (119), macrophages (120), and microglial cells (121). All of these findings suggest that TRPV4 is a receptor for LPS recognition independently of TLR4 (122). Furthermore, TRPV4 is abundantly expressed in pulmonary microvascular endothelial cells (123), during pulmonary inflammation, TRPV4 is highly expressed and up-regulated in vascular endothelial cells, suggesting that TRPV4 is associated with the development of infection-associated acute lung injury (124). The current study suggests that pharmacologic inhibitors of TRPV4 can reduce the production of proinflammatory cytokines, restore vascular endothelial cell function, and decrease sepsis-associated hyperinflammatory response and mortality in the septic rat (124). Ca^2+^ endocytosis evoked by TRPV4 activation in vascular endothelial cells generates a large amount of calcium signaling concentrated locally, usually confined to within a few micrometers of the channel (125), Therefore, how does overactivation of TRPV4 lead to cytoplasmic calcium-dependent events throughout the cytoplasm when TRPV4 is overactivated? The endoplasmic reticulum (ER) is the major Ca^2+^ reservoir in the cell. Inositol 1,4,5-Trisphosphate Receptors, IP3Rs, are the main channels for calcium release from the endoplasmic reticulum and play a key role in calcium signaling regulation in endothelial cells (126). TRPV4 has a binding site to the IP3Rs in a second ANK structural region at the C-terminus (AA 812-831). TRPV4-mediated Ca^2+^ influx can trigger the activation of type 1 1,4,5-Trisphosphate Receptors and initiate the re-release of Ca^2+^ in the form of a spreading wave, thereby magnifying the initial calcium signal, which is a process known as Calcium Induced Calcium Release (CICR) (125). Furthermore, IP3R1 can lead to sustained activation of TRPV4, resulting in continued enhancement of the CICR response (127), ultimately contributing to intracellular calcium overload. Taken together, calcium-induced calcium release mediated by TRPV4 interacting with IP3R1 is a critical mechanism that leads to calcium overload in pulmonary microvascular endothelial cells.

Furthermore, as equally significant physiological cells and immune barriers in the lungs, epithelial cells and alveolar macrophages, we also looked into the function of calcium overload in these cells. Two subtypes of alveolar epithelial cells (ATI and ATII) exhibit significant expression of TRPV4, which mediates calcium influx and ultimately exacerbates pulmonary edema (128). When calcium sensitive receptors (CaSRs) are activated in alveolar macrophages (AMs), they trigger pyroptosis of AM cells, initiate the NLRP3 inflammasome pathway, and enhance the mortality rate of acute respiratory distress syndrome (129). Research shows that the resolution of inflammation and bacterial clearance in ARDS can be facilitated by macrophages through the inhibition of calcium channel orai1 mediated calcium influx (130).

Although prior research has demonstrated the essential function that calcium overload plays in sepsis-induced acute lung injury, the exact mechanism of action of this effect is still unknown. Some results continue to support the idea that calcium overload can induce ferroptosis in ALI, despite the fact that mechanistic study on this relationship has not yet been conducted in the field. Previous studies have shown that calcium overload can indeed lead to mitochondrial dysfunction and ferroptosis (22). An analysis of the literature showed that in patients with critical COVID-19, increased tissue calcium in-flow in the lungs and endothelium was closely associated with the adverse outcome of ultimately occurring ferroptosis (131). Guo et al. found that PIEZO1, a calcium channel that is highly expressed in pulmonary tissues, mediates ionizing radiation-induced ferroptosis in pulmonary endothelial cells by increasing intracellular calcium concentration and calpain activity (83). Thus, based on previous work, we hypothesize that LPS causes calcium overload in lung effector cells via the interplay of TRPV4 and IP3R1 in sepsis-induced acute lung injury. Overabundance of Ca^2+^ increases the development of ALI by promoting lipid peroxidation, initiating calcium signaling cascades, inducing ferroptosis in lung cells, and rupturing the lung’s circulatory-immune barrier.

## 6 Notice the ways that calcium signaling affects ferroptosis: a novel approach to managing acute lung injury

The ferroptosis process is a gene expression event that involves precise regulation of many components. Any medication or small molecule substance that targets a particular link in the ferroptosis process may have an impact on the advancement of ferroptosis. For several ferroptosis-related disorders, ferroptosis agonists like ML210 and RSL3 and inhibitors like Fer-1 and Lip-1 are being researched (17). Furthermore, in response to the disruption of iron homeostasis during ferroptosis, the development of “iron chelators” has opened up new avenues for the treatment of various illnesses. DFO, for instance, can be used to treat neurological illnesses and sickle cell anemia, but it may also have harmful side effects such edema and anemia (132). The development of novel medications is still hampered by a number of issues, including poor pharmacokinetics and frequent side effects. As a result, the majority of current research on medications used to cure ferroptosis occurs in preclinical animal models, with relatively few entering clinical usage. Thus, the development of novel, potent therapeutic medications to control ferroptosis is a critical problem that requires immediate attention in the present. As a crucial second messenger in cells, calcium ions can take part in a number of biological processes related to ferroptosis and may also be involved in sepsis-induced acute lung injury. Recent studies on the roles of calmodulin and calcium ion channels have revealed possible targets for novel medication development. The use of calcium homeostasis regulators to ferroptosis in ALI, however, is still largely unexplored. In conclusion, research on calcium homeostasis is an intricate and important field that will further our knowledge of the processes behind intracellular calcium ion balance and its significance in many illnesses. It is theoretically feasible to concentrate on the role that calcium homeostasis plays in ferroptosis in ALI.

### 6.1 Regulating sepsis-induced acute lung injury by focusing on calcium homeostasis

An increase in Ca^2+^ influx can enhance endothelial permeability and contribute to neutrophil inflammatory responses, resulting in the loss of lung tissue shape and function as well as the spread of inflammatory consequences. Understanding the mechanism of calcium homeostasis disruption may thus be useful in predicting ALI early on. As an example, Tian et al. emphasized the potential of PAD2, a member of the calcium-dependent enzyme family of peptidylamino acid deiminases (PADs), as a therapeutic target and indicating sign for septicemia ALI (133). Recent study suggests that inhibiting the influx of Ca^2+^ into effector cells is another viable therapeutic option for ALI. Focusing on the regulating mechanism of calcium homeostasis is probably beneficial. Below are some fundamental studies on targeting calcium homeostasis for therapeutic goals in sepsis-induced acute lung damage. Extracellular vesicles produced from endothelial cells (EnCs) and type II alveolar epithelial cells (EpCs II) might inhibit sepsis-induced ALI by modulating the RGS1-mediated calcium signal-dependent immunological response (134). Pulmonary microvascular endothelial cells express T-type calcium channels (Cav3.1) (135). A basic study revealed that two traditional T-type calcium channel blockers, Mibefradil and Flunarizine, can successfully treat LPS-induced ALI (136). Hederasaponin C, an active ingredient of a traditional Chinese medicine, regulates calcium homeostasis via the PIP2/NF-κB/NLRP3 signaling pathway, alleviating LPS-induced ALI (137). It is clear that calcium homeostasis is involved in the development of ALI, and decreasing intracellular calcium overload has therapeutic utility for sepsis-induced ALI. As we all know, the regulatory mechanism of intracellular calcium homeostasis is extremely complex, involving numerous calcium channels, enzymes, receptors, and ion interactions. The negative effects of calcium homeostasis imbalance on ALI are multifaceted, affecting not only cell death but also the body’s circulation, immunity, energy metabolism, and so on. It has the potential to aggravate the patient’s condition as well as alter the long-term prognosis. The control mechanism of calcium homeostasis in ALI is currently being studied at a basic level. If new low-cost and effective targeted medications can be created in the future, patients and economic burden will be significantly reduced.

### 6.2 Regulating ferroptosis by focusing on calcium homeostasis

**Figure 3:**
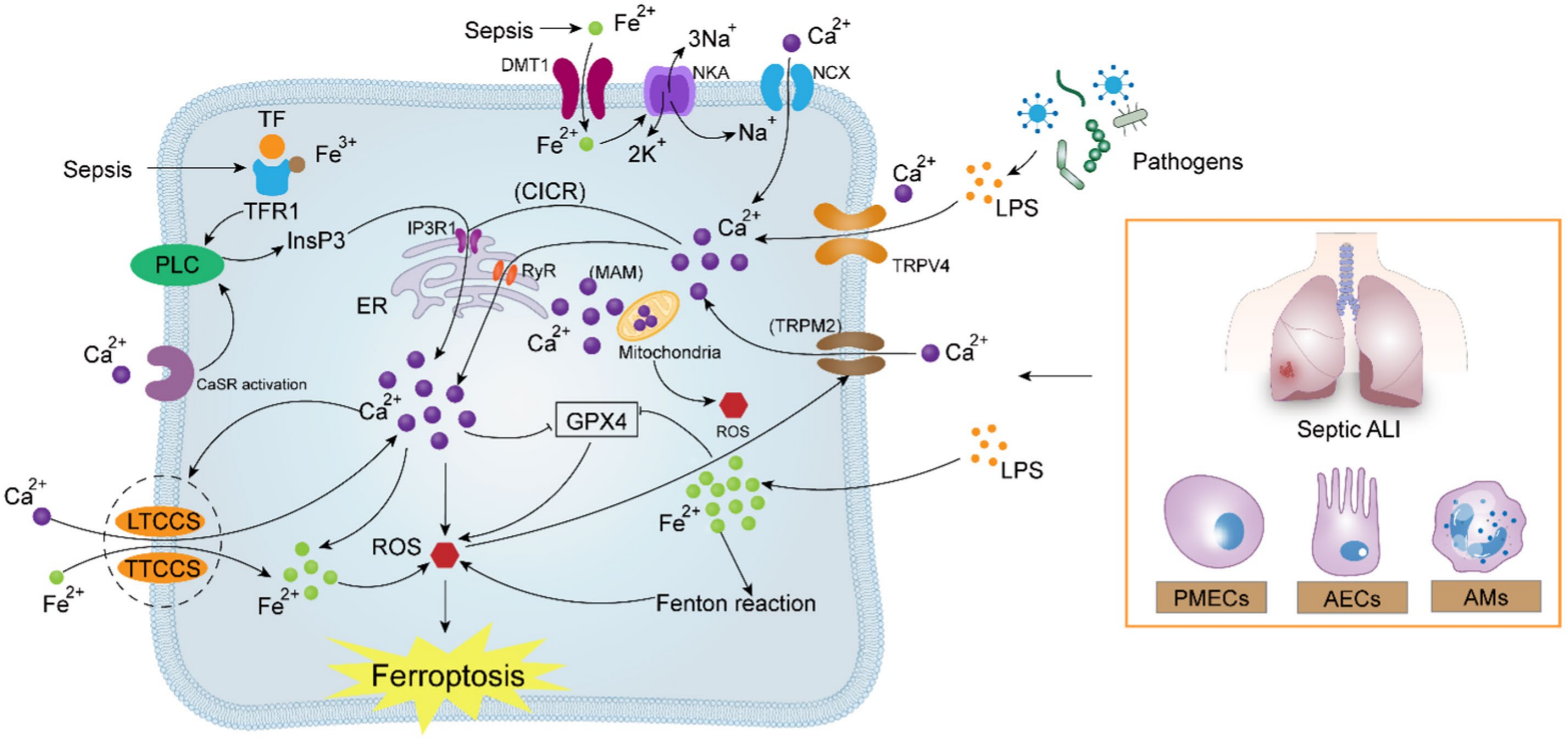
Potential correlation between calcium signaling and ferroptosis in sepsis-induced acute lung injury. In sepsis, pathogenic microorganisms generate high amounts of LPS, which activate Ca^2+^ uptake by the TRPV4, TRPM2, IP3R1, and RyR channels, leading to intracellular calcium overload via the “CICR” reaction. Calcium overload aggravates the existing excessive load of Fe^2+^ under oxidative stress, reverses NCX and NKA, and simultaneously activates the attraction of LTCCs and TTCCs to Fe^2+^. The crosstalk between Ca^2+^ and Fe^2+^ forms a positive feedback mechanism, which ultimately contributes to the intracellular “ROS storm,” oxidizing the cell membrane and aggravating ferroptosis.

The advantages of managing ferroptosis in sepsis-induced acute lung injury are undeniable, as addressing calcium homeostasis has an excellent effect on reducing ferroptosis. By treating calcium overload during sepsis with calcium homeostasis regulators, organ dysfunction in sepsis may be prevented. Wang et al. discovered through experimentation that the decrease in calcium ions flowing through the LTCCs may be the mechanism by which clovirobuxine D prevents ferroptosis in septic cardiomyocytes (138). Xie et al. found that the inhibition of ferroptosis in septic neurons by ferritin-1 may be associated with the reduction of activation of the calcium transport proteins PLCG and PLCB (139). In ALI, endothelial inflammation can be successfully inhibited by certain calcium channel blockers. A recent study by Song et al. revealed that the calcium channel blocker, limerizine, alleviated LPS-induced acute lung injury in mice (140). Hao et al. summarized the therapeutic potential of some calcium antagonists in inhibiting pulmonary endothelial permeability in ALI (107). According to a recent study, the endoplasmic reticulum-localized protein MS4A15 depletes stored Ca^2+^ and contributes independently to resistance to ferroptosis. Overexpression of the MS4A15 dramatically alters Ca^2+^ homeostasis and inhibits the expression of IP3R1, contributing to the extensive lipid remodeling (141). In another work, Gleitze et al. summarized the important association of calcium and iron in neuronal death mediated by ferroptosis, suggesting that restoring the normalization of the calcium signaling pathway is the likely route to rescue neurons from ferroptosis (142). According to a different studys, several natural remedies that control calcium homeostasis can regulate ferroptosis. These natural remedies are frequently easy to use, affordable, and have few negative effects. For instance, the natural substance eryanin found in Dendrobium officinale targets the Ca^2+^/CaM signaling pathway to cause ferroptosis, which is beneficial in causing the death of lung cancer cells (101). Saikosaponins B2 (SB2), the active component of traditional Chinese medicine, controls intracellular calcium homeostasis to prevent ferroptosis and endoplasmic reticulum stress (143). These findings confirm the hypothesis of a mechanistic relationship between ferroptosis and calcium homeostasis in sepsis-induced ALI and show the potential of calcium homeostasis regulators in the treatment of acute lung injury in sepsis. We think there should be a stronger, positive correlation between ferroptosis and calcium homeostasis in ALI. Thus, we urge scientists studying natural medicines and related compounds to focus on the regulatory importance of calcium homeostasis in ALI for ferroptosis. To provide evidence in support of therapeutic applications, more thorough investigation is required to determine the precise mechanism of their interaction (Figure 3).

## 7 Discussion

The normal physiological function of the lungs involves the coordinated action of various cells, such as alveolar epithelial cells, pulmonary microvascular endothelial cells, alveolar macrophages, and so on, to maintain the lungs’ circulatory immune barrier. Sepsis introduces an overwhelming burden of microorganisms, making the lungs, which are already delicate organs, vulnerable to irreparable damage that is both quick and severe. As a result, sepsis-induced acute lung injury has a high death rate and is becoming an increasingly serious threat to human health. However, there is still a paucity of effective treatment methods, necessitating the discovery of new molecular bases for new medications and treatments.

Ferroptosis is a sequence of precisely orchestrated gene expression processes, and while the aforementioned programs mostly function at the endpoint of cell death, they also serve as crucial defensive mechanisms, actively eliminating damaged cells for the body. As a consequence, ferroptosis, which regulates the number and activity of lung cells, could be a significant target for preventing acute lung injury in sepsis. Lipid peroxidation is a process of reactive oxygen species oxidation of biological membranes that occurs after increasing oxidative stress. It is accompanied by the formation of a high number of lipid peroxidation products, which causes cell and organ damage. Fe^2+^ overload and excessive ROS generation constitute essential components of the ferroptosis process, with lipid peroxidation being the end consequence. The excessive generation of ROS may lead to cell ferroptosis (144). Ca^2+^, an important second messenger in cells, perform a vital part in sepsis, which is mostly expressed as intracellular calcium overload (19, 20). In this article, we reviewed the regulatory mechanisms of ferroptosis, identified putative mechanisms of ferroptosis-induced septic ALI, and examined the function of intracellular calcium homeostasis in ferroptosis. We hypothesize that intracellular calcium overload promotes ferroptosis, which exacerbates sepsis-induced ALI. Firstly, in sepsis, intracellular calcium overload can cause ROS generation via a variety of mechanisms (76), boosting lipid peroxidation and accelerating cell ferroptosis. Secondly, calcium homeostasis regulates the body’s redox system, as well as lipid synthesis and metabolism (82, 88). Finally, there is already a strong link between intracellular Ca^2+^ and Fe^2+^, particularly in the context of sepsis. Calcium ions increase the entry of Fe^2+^ into cells via different channels or proteins, and both calcium and iron have roles in ROS production, mitochondrial and endoplasmic reticulum function, and alveolar structural stability in ALI (98, 99). As a result, addressing calcium homeostasis to regulate ferroptosis is a possible treatment approach for ALI. In response to the aforementioned molecular pathways, we have presented an approach for treating ferroptosis in sepsis-induced acute lung injury by targeting calcium homeostasis, with a focus on the discovery and use of natural therapies. Some natural compounds found in plants and traditional Chinese medicine can influence calcium homeostasis and iron death via distinct processes. They offer the advantages of being low-cost and having few adverse effects, making them worthy of future development. Sepsis, unlike other chronic diseases, is a serious condition that can impair the body’s microenvironment homeostasis. Electrolyte imbalances, such as hypocalcemia, are widespread during sepsis and pose significant annoyance to healthcare staff. However, we recommend that more researchers investigate the significance of intracellular calcium homeostasis imbalance in sepsis, since this could signify a deeper pathological mechanism in the body. Compared to other organs, the lungs are more vulnerable to sepsis damage. Thus, understanding the mechanism of calcium homeostasis in ferroptosis is critical for lung protection in sepsis. This also raises the question of whether calcium homeostasis is linked to other regulatory cell demise. For example, cell pyroptosis and autophagy merit additional investigation.

This article summarizes and generalizes previous research, and then expands and suggests theories, offering fresh references for mechanism study and medication treatment of sepsis-induced ALI. Is there a more finely tuned molecular mechanism linking calcium homeostasis and ferroptosis? Is there a more targeted medicine available? More basic and clinical research is needed in the future to back up our speculation.

## 8 Conclusion and perspective

In summary, the interconnection between Ca^2+^, iron, and ferroptosis could have a major part in the pathogenesis of septic ALI, and current studies on the relationship between the three mainly focus on the oxidative stress, lipid peroxidation, inactivation of the GPX4 system, as well as the interaction between Ca^2+^ and Fe^2+^. Accordingly, relevant therapeutic and biomarker predictions for targeting ferroptosis in septic ALI in response to abnormal intracellular calcium signaling and iron homeostasis imbalance demonstrate certain diagnostic and therapeutic value, however, detailed basic experimental investigations are still lacking. In the future, developing novel targets and directions for the development of relevant indicators and therapeutic treatments could entail further elucidation of the molecular biological relationship between calcium signaling and ferroptosis in septic ALI.
